# Impact of inclusive leadership on employee innovative behavior: Perceived organizational support as a mediator

**DOI:** 10.1371/journal.pone.0212091

**Published:** 2019-02-28

**Authors:** Lei Qi, Bing Liu, Xin Wei, Yanghong Hu

**Affiliations:** 1 School of Management, Shandong University, Jinan, P. R. China; 2 Kings College, The University of Aberdeen Business School, Old Aberdeen, Aberdeen, Scotland, The United Kingdom; University of Calgary, CANADA

## Abstract

Despite extensive literature on leadership and its impact employee innovative behavior, few studies have explored the relationship between inclusive leadership and employee innovative behavior. To address this gap, this study aimed to investigate how inclusive leadership influenced employee innovative behavior by examining perceived organizational support (POS) as a mediator. We used multi-wave and multi-source data collected at 15 companies in China to test our theoretical model. Results revealed that inclusive leadership had significantly positive effects on POS and employee innovative behavior. Furthermore, POS was positively related to employee innovative behavior and partially mediated the relationship between inclusive leadership and employee innovative behavior. We discussed implications and limitations of this study as well as avenues for future research.

## Introduction

In a competitive environment characterized by globalization, shortened product life cycles, and rapid technological change [1], innovation has been regarded as the crucial facilitator for growth, performance, and competitiveness [2]. Managers and scholars have increasingly emphasized the important influence of innovation on competitive advantage, sustainable development, and long-term organizational success [3]. A key issue of innovation was that an individual had an innovative, novel and creative idea and developed that idea beyond its initial state [4]. Given that employee innovation in organizations was of critical importance to an organization, it was vital to identify factors that could stimulate employee innovative behavior [5], which referred to employee’s engaging innovative activities [6,7].

Investigators have sought to identify determinants of employee innovative behavior [6,8]. Many determinants have been explored, such as knowledge sharing [9], human resource (HR) management practices [10], innovation climate [11], absorptive capacity [12], and perceived innovation job requirements [7]. Among all these determinants of innovative behavior, leadership has been arguably noted as the most important factor that influenced creativity and innovation in organizations [13]. Several studies indicate that transformational leadership [1,14], ethical leadership [15], and paternalistic leadership [16] significantly influenced employee innovative behavior. Despite those studies has not yet examined the effect of inclusive leadership on innovative behavior. Inclusive leadership was quite different from other kinds of leadership [17]. Inclusive leadership closely matched the determinants of innovation at the workplace, some of which were inclusiveness, openness, uniqueness, and support for innovation [17,18]. Studying the impact of inclusive leadership on innovative behavior could provide further insights into extant literature regarding the effect of leadership on innovative behavior. Unfortunately, published empirical studies on the link between inclusive leadership and innovation performance are rare.

The current study developed novel theoretic insights on how employee innovative behavior was affected by inclusive leadership. Innovation involved change [19], which by its nature (i.e. diversity) required inclusiveness, openness, and support. Business organizations capable of fostering an innovation-supportive work environment may realize a sustainable competitive advantage in innovation [20]. To examine the relationship between inclusive leadership and employee innovative behavior, we proposed employees’ perceived organizational support (POS) of employee, as a mediator for this relationship. POS, which was valued as assurance that would be available from the organization when needed to carry out their job effectively and deal with stressful situations [21,22]. Researchers have highlighted the importance of inclusive leadership [17] and organizational support [23] in stimulating employee innovative behavior. However, few studies focused on the relationship between inclusive leadership and employee innovative behavior through employee’s POS [24].

In the current study, we contributed to extant research [17,23] by investigating the influence of inclusive leadership on employee innovative behavior through POS. Our study aimed to make two major contributions to understanding the role of leadership behavior in developing organizations’ competitive advantage [25,26]. First, our study contributed to research on organizational leadership by emphasizing the role of inclusive leadership, as “research into inclusion is still in its infancy” [27]. Second, our study extended previous studies on the antecedents of employee innovative behavior by examining how organizational contextual factors such as inclusive leadership (Time 1) and POS (Time 2) influenced employee innovative behavior (Time 2). This research echoed the call for “future studies that may adopt a longitudinal approach to study the effect of change in supervisors’ leadership style on employee creativity” [11]. Also, this study responded to call for rich and nuanced conceptual research in the innovation field, especially concerning the role of employees’ cognition in motivating their innovative behavior.

## Theoretical Foundation and research hypotheses

### Inclusive leadership

The concept of inclusive leadership was originally proposed in the field of management by Nembhard and Edmondson (2006) [24], which was defined as the “words and deeds by a leader or leaders that indicate an invitation and appreciation for others’ contributions.” Subsequently, Hollander (2009) [28] defined inclusive leadership as a win-win situation with a common goal and vision of interdependent relationships. Hollander emphasized the important role of followers in this relationship and paid attention to their perception of leadership. Ospina (2011) [29] described an inclusive leader as valuable, and someone who accepted staff at all levels in the organization and was responsible for results. Furthermore, an inclusive leader was considered as playing a key role in forming an inclusive organization. Specifically, inclusive leadership contained three dimensions: (1) Leaders tolerated employees’ views and failures by listening attentively to their views, rationally tolerated their errors, and provided encouragement and guidance to support staff when they make mistakes. (2) Leaders recognized and trained employees by respecting and focusing on employee training and praising achievements rather than displaying jealousy [30]. (3) Leaders treated employees fairly, considered their needs and interests, showed a fair attitude towards employees, and ensured that they share earnings.

**Fig 1:**
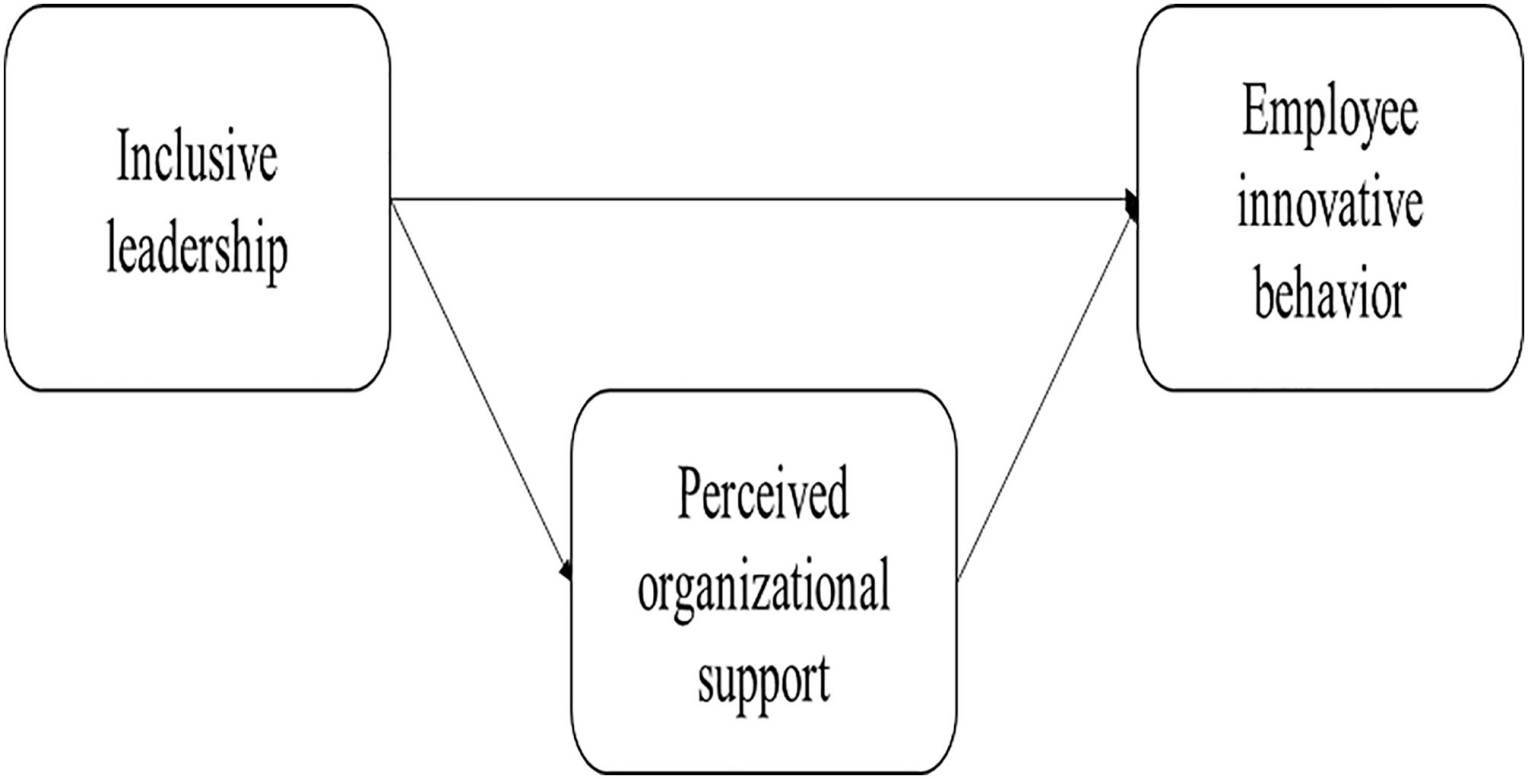
Research model.

Compared with the other forms of leadership that may be conceptually related, inclusive leadership held unique nature of acceptance, belongingness, uniqueness, and inclusiveness [18]. Specifically, transformational leadership focused on motivating and developing employees based on the organization’s needs [31], and transformational leadership centered in the leader, without active employee involvement, reciprocal influence, and rewards [28]. In contrast, inclusive leadership emphasized on accepting employees for who they were, allowing them to contribute their unique abilities and views, and encouraging them to involve in organizational activities. Although, servant leadership focused on helping employee grow and succeed [32], inclusive leadership focused on tending to member’s needs for work group openness and availability. While empowering leadership focused on leading by example, sharing power, teaching and coaching [33] inclusive leadership facilitated the perception of inclusiveness and accessibility. Based on above differences, the overlap between inclusive leadership and existing conceptualizations of leadership was minimal, and other types of leadership could not fully capture key tenets of inclusive leadership [18]. Despite the unique and critical role of inclusive leadership in the leadership research, to date, few studies have investigated the link between inclusive leadership and employee innovative behavior. The aim of this paper was to investigate how supportive conditions in an organization affected employee innovative behavior through POS. Fig 1 showed the research model developed for this purpose.

### Employee innovative behavior

Innovative behavior was considered as a series of activities pertaining to idea generation, idea promotion, and idea realization for new technologies, processes, techniques, or products [34–36]. Employee innovative behavior focused on the innovation process, (i.e., engaging in innovative activities) rather than the innovation outcome (i.e., new products) [6,37], which was beyond the concept of creativity [7]. In this study, we followed Shin, Yuan, and Zhou’ (2017) research, “draw from the literature about innovative behavior in general, including the literature on creativity”, to develop our theoretical model [7]. For decades, several types of leadership have been demonstrated to influence employee innovative behavior in organizations [38]. Amabile et al. (1996) proposed that freedom, supervisory encouragement, and organizational support were closely related to innovation [39].

From theoretical perspectives, inclusive leadership can stimulate employee innovative behavior in multiple ways. First, inclusive leaders can energize employees to engage in innovative process [40]. Conger and Kanungo considered inclusion as a process of improving internal perception of organization employees and as a concept related to intrinsic motivation [41]. Increasing motivation leaded to more involvement in innovative behavior [40,42]. Second, based on organizational support theory [43], employees’ work outcomes relied on organizational support. Inclusive leaders were able to provide resources including information, time, and support necessary for innovative behavior [44]. “Leader inclusiveness is directed toward encouraging and valuing the different viewpoints of diverse members within team interactions” [27]. When employees were supported by their leader, they would get more autonomy and freedom to engage in innovative behavior [45]. Boren argued that inclusion was based on employees’ basic trust, explaining that managers use various skills to improve the capacity and potential of subordinates’ behavior [46]. In addition, Randel et al (2018) stated that the inclusive leadership might facilitate organization employees perceiving belongingness (by supporting team members, ensuring justice and equity, and sharing decision-making) in the organization while maintaining their uniqueness (by encouraging diverse contributions and helping team members fully contribute) within the organization as they fully contribute to the organization processes and outcomes of innovation (i.e. creativity) [18]. Third, inclusive leaders could serve as role models for innovative behaviors [47]. Nembhard and Edmondson suggested that leader inclusiveness was positively related to engagement in quality improvement work [24]. Carmeli et al. argued that inclusive leadership demonstrated a specific relationship exhibited through openness and harmony in communication, accessibility, and offering [17]. Through appropriate inclusiveness, leaders created an environment where employees had a greater sense of responsibility [48], had more decision-making autonomy, and received more information and feedback as well as support and encouragement [49]. General openness, availability, and accessibility facilitated employee involvement in innovative work [17]. Innovative behavior was often noted as “discretionary behavior” [34]. Inclusive leadership’s unique features reshaped followers’ perception of support and enhanced more innovative behavior [18]. Thus, based on organizational support theory [43], we formulated the following hypothesis:

**Hypothesis 1:** Inclusive leadership is positively related to employee innovative behavior.

### Perceived organization support

Eisenberger and Stinglhamber proposed that POS referred to "employees develop global beliefs concerning the extent to which the organization values their contributions and cares about their wellbeing" (1986: 501) [43]. POS was grounded in the theory of organizational support, underlining the importance of viewing employees as valued organizational assets [23]. New areas of research emphasized organizational support as an important factor affecting employees’ willingness to contribute to the organization [50]. Inclusive leadership represented an important organizational aspect that can assist in creating a more innovation-supportive work environment [17,51], but our conceptual understanding of the supportive mechanisms linking inclusive leadership to employees’ innovative behavior remains underdeveloped. In this section, the mediating role of POS will be clarified. How inclusive leadership affects POS will be clarified first, and subsequently the effect of POS on innovative behavior will be explained.

According to organizational support theory [43], employee’s perception of favorable treatment received from the organization, such as supervisor support, should increase POS [52]. Supervisors in leadership roles played a key role in providing organizational resources and rewards for subordinates, and therefore, should be regarded as an important source of organizational support [53]. Inclusive leader could provide benefits that subordinates could make use of. Supportive behaviors from inclusive leader helped subordinates perceive that their contributions were valued and their well-being were cared about [54], and should enhance POS. Thus, supportive behaviors from inclusive leaders should be closely related to POS, and we proposed the following hypotheses:

**Hypothesis 2:** Inclusive leadership is positively related to POS.

Employees’ innovative behavior can be stimulated by fostering a work context in which employees feel supported to generate, promote, and realize inventive ideas and concepts [55]. Innovation and spontaneous problem solving may additionally be associated with perceived support [55, 56]. First, on the basis of social exchange theory [57], POS should elicit the norm of reciprocity, leading to employee’s obligation to help the organization to achieve its goals, as well as the expectation that increased efforts on the organization’s behalf would be noticed and rewarded [52]. Employees who perceived high levels of POS were more likely to feel a duty of caring for the organization’s development and help it achieve its goals. Luksyte and Spitzmueller (2016) indicated that “high levels of POS create a sense of obligation to contribute to the organization” [23]. This responsibility enhanced employees’ affective commitment to the organization and the will of retention. For instance, according to the social exchange theory (the principle of reciprocity), employees’ sense of responsibility and emotional commitment would help decrease absenteeism and increases altruistic behavior [58]. Barksdale and Werner (2001) argued that POS could motivate employees to better fulfill in-role behavior [59]. Similarly, employees who felt more POS experience a better needs–supplies fit, and increased creativity [23]. Also, Bammens claimed that organizational care, which was closely related to POS, positively predicted innovative behavior [20]. Conversely, when employees perceived low support of organization, their involvement in innovation would be lessened [55]. Therefore, we suggest the following hypothesis:

**Hypothesis 3:** POS is positively related to employee innovative behavior.

Inclusive leadership could influence employee innovative behavior through POS. According to social exchange theory [57], the more desired inclusion, permission, and resources employees received from the leader and organization, the higher the POS and the more motivated and obliged they were to reciprocate by being more engaged in innovative behaviors. Employees were highly attuned to leaders’ behavior and examined leader actions for information on what was expected and acceptable in organizational interactions [60]. To assist them in unleashing their innovation potential, employees may require a general sense that leaders showed support [4,51]. On the one hand, if leaders care about subordinates’ needs and feelings, provided positive feedback, encourage employees to express their own ideas and develop their skills, and helped them solve job-related problems, organizational loyalty and employees’ interest in work will greatly improve, increasing their enthusiasm to explore and innovate [61]. Furthermore, inclusive leadership could inspire a sense of responsibility: When employees received support from the organization, their confidence increases, they demonstrate more voluntary acts, and actively use their potential to fulfill the organization’s mission, further increasing their abilities and awareness of innovation [62].

If a leader was inclusive and welcomed challenges, employees were likely to perceive more organizational support and became more innovative, for perceived support for innovation was an important source of innovation or creativity [63]. Leadership can affect innovative behavior through its influence on employees’ perceptions of a climate supportive of innovation [1]. Inclusive leaders, by intellectually stimulating their subordinates, championing innovation, and tolerating attempts, helped establish a climate that employees felt encouraged and energized to explore innovative approaches in their work. Fitzpatrick claimed that the best way to support progressive care nurses was inclusive leadership [64]. Inclusive leadership delivered to employees the unique perception of support from leaders that should increase employee innovative behavior. Taken together, POS increased the likelihood that employees would develop more creative solutions and approaches to address issues [55]. Hence, we proposed the following hypothesis:

**Hypothesis 4:** POS mediates the relationship between inclusive leadership and employee innovative behavior.

**Fig 2:**
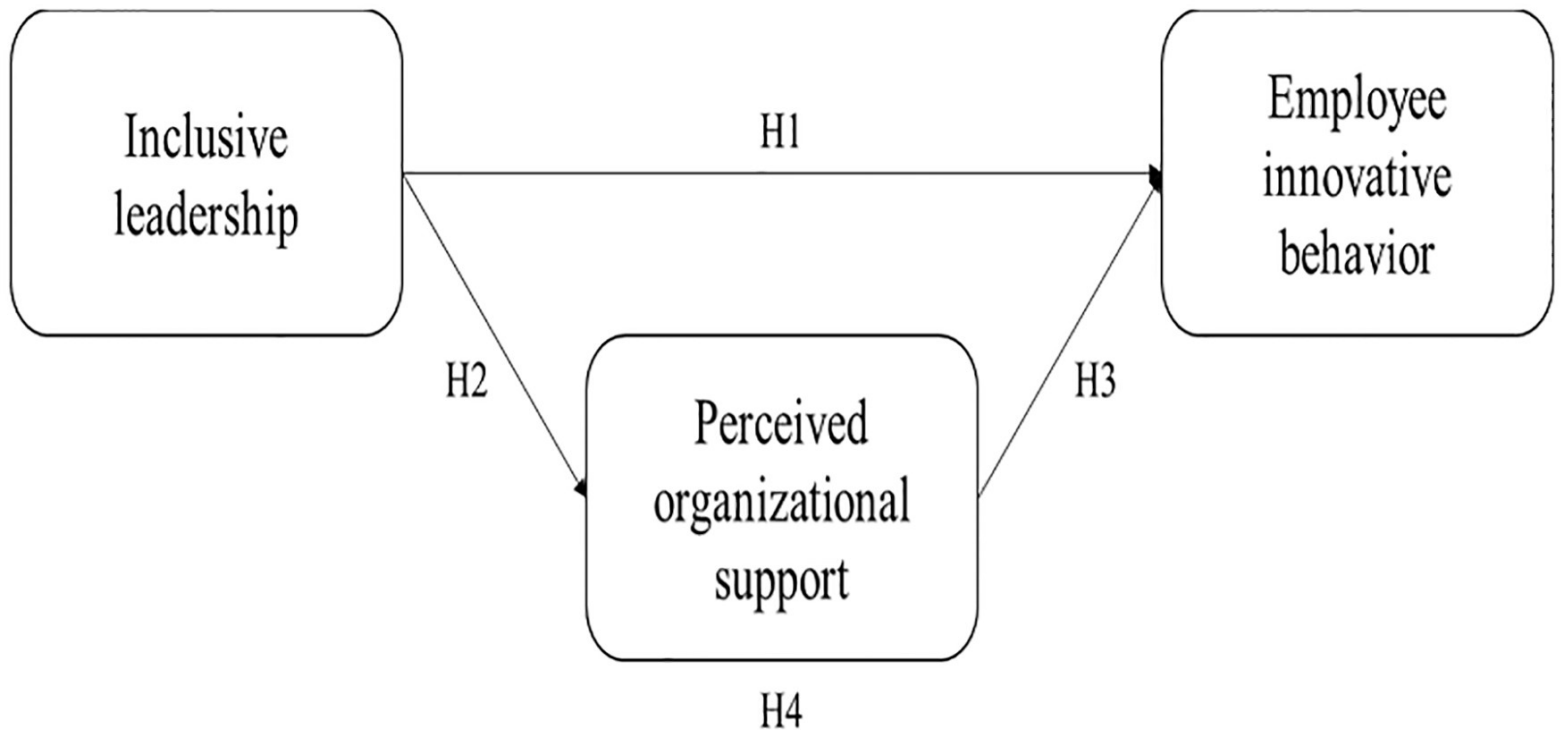
The hypothesis model.

The hypothesis model of our study is illustrated in Fig 2.

## Research design

### Sample and procedures

Data were collected among employees and supervisors from 15 service-based organizations such bank, law offices, Sinopec, and retailing stores in 6 cities in China. Initial contacts with these companies were via connections to current and former MBA students. We used three ways to reduce the common bias [65]: First, a two-wave survey with a three-month interval was conducted. Using different time intervals for data collection helped to minimize the impact of memory and common method variance bias and enhanced the robustness of findings by collecting data on predictors and outcomes separately [65]. Second, a two-source survey with employee-supervisor matched was adopted, because employee innovative behavior rated by supervisors was much more valuable. Third, during the process of questionnaire design and distribution, strict program control was carried out in this study. Each survey was conducted with the help of human resources department. At the beginning of the survey, 401 subordinates and their matching supervisors were randomly selected, and all employees had the freedom to decide whether to participate in this study. Every participant received a red pocket with a random amount of money (5–20 RMB) for each questionnaire completed. To ensure participants’ confidentiality and decrease their fear of exposure and risk of liability, surveys were placed into a sealed envelope and respondents were instructed to return the completed questionnaires directly to the researchers. Anonymity was assured. The survey questionnaires were coded before being distributed.

Time 1 (T1), we administered questionnaires to 401 employees in 127 teams, who were asked to provide their demographic information (e.g., age, gender, education, and tenure) and perceived inclusive leadership. We received usable responses from 364 employees in 116 teams, for a response rate of 90.77%. Three months later, we conducted the second survey (T2), 330 employees who participate in the T1 survey and 112 supervisors were available. Employees were asked to report their perceived organizational support and supervisors were asked to rate their subordinates’ innovative behavior. 329 questionnaires reported by employees and 105 questionnaires rated by supervisors were collected. As a result, we obtained completed questionnaire from 226 employees (a response rate of 56.36%) and 75 matched supervisors (a response rate of 66.96%) after excluding missing data. Of the sample, 61.10% of the respondents were female; most (81%) were below 35 years old; 54% employees got bachelor degree, 41.20% employees got college degree or below, 4.80% employees got master degree or above; the tenure of employees ranged from 1 year to 10 years (40.7% of employees worked 1–3 years, 18.6% of employees worked 4–6 years, 18.1% of employees worked 7–10 years).

### Ethics approval

An ethics approval was not required as per institutional guidelines and national laws regulations because there’s no unethical behaviors existing in the research procedures. We just conducted questionnaire survey and were exempt from further ethics board approval since our research did not involve human clinical trials or animal experiments. Also, the content of the questionnaire did not involve any sensitive or personal privacy or ethical and moral topics. In the first page of the questionnaire, information on consent procedures was included and participants were notified that consent was to be obtained by virtue of survey completion. Meanwhile, we informed that participants about the objectives of the study and guaranteed their confidentiality and anonymity. The way to fill in the questionnaire is to take out the secret system, which can further ensure rights of people who answer the questionnaire. All the participants were completely free to join or drop out the survey. Only those who were willing to participate were recruited.

### Measures

To maximize the validity and reliability of the measurement tools, we used existing scales published in top journals. The original scales were all written in English. Thus, we followed the back translation procedures recommended by Brislin (1980) [66] to translate the measures. A management scholar who was fluent in both English and Chinese translated the items from English into Chinese. We then asked another bilingual management professor to translate the items from Chinese back into English. We also asked a management researcher to check the English and Chinese translations, and any discrepancies in the translation procedure were solved through discussion. Furthermore, before finalizing the formal questionnaire and survey, a pre-survey was conducted to guarantee the appropriateness of the questionnaire design and diction to the study context. The questionnaire was then revised based on feedback regarding the pre-survey. Unless otherwise noted, we used a five-point Likert scale ranging from “1 = totally disagree” to “5 = totally agree” to assess each measure.

**Inclusive Leadership:** We assessed inclusive leadership using a nine-item scale that were developed by Carmeli et al. (2010) [17] (T1, rated by employee). A similar approach was used by Hirak, Peng, Carmeli, and Schaubroeck (2012) [67] and Choi, Tran, and Kang (2016) [68]. A sample item was: “The manager is open to hearing new ideas.” The reliability coefficient (Cronbach’s Alpha) of the scale in this study was 0.927, indicating that the scale has good reliability.

**Perceived organizational support (POS):** The eight-item scale by Eisenberger (1986) [43] was employed to measure POS (T2, reported by employees). The scale has been used by Edwards and Peccei (2015) [69] and Zumrah and Boyle (2015) [70]. A sample item was: “My organization takes pride in my accomplishments at work.” The reliability coefficient (Cronbach’s Alpha) of the scale in this study was 0.952, indicating that the scale had good reliability.

**Innovative behavior:** The nine-item scale by Janssen (2000) [34] was used to measure employee innovative behavior (T2, rated by supervisor). The scale had been used by Janssen (2003) [58] and Agarwal, Datta, Blake-Beard, and Bhargava (2012) [71]. The scale measured employee innovative behavior based on three aspects of “ideas put forward,” “promotion of ideas,” and “apply the idea of.” A sample item was: “Creating new ideas for difficult issues (idea generation).” The reliability coefficient (Cronbach’s Alpha) of the scale in this study was 0.951, indicating that the scale had good reliability.

**Control variables:** Based on previous research [72–74], we selected gender, age, education, and tenure (which reflects work domain expertise) [75,76] as main control variables for their probable association with employee perceived organizational support and innovative behavior.

## Results

### Descriptive statistics

Table 1 presented descriptive statistics and correlations. Consistent with the hypotheses of this study, as shown in Table 1, inclusive leadership was positively related to employee innovative behavior (r = 0.302; p <0.01) and POS (r = 0.697; p <0.01), and POS was positively related to employee innovative behavior (r = 0.279; p <0.01).

**Table 1:** Means, standard deviations, and correlations between main variables.

Variable	Mean	SD	1	2	3	4	5	6	7
1. Gender	.438	.572	1						
2. Age	2.518	.605	-.234**	1					
3. Education	1.783	.575	-.210**	-.034	1				
4. Tenure	3.084	1.239	.029	.368**	-.105	1			
5. Inclusive leadership	4.441	.600	.154*	-.002	-.047	.076	1		
6. POS	4.365	.730	.112	.070	-.046	.102	.697**	1	
7. Innovative behavior	3.860	.806	.238	.182**	-.173**	.264**	.302**	.279**	1

Note: N = 226,

** p <0.01,

* p <0.05 (two-tailed)

### Tests of hypotheses

To test hypothesized main effects and mediation effect, we followed Hayes’ approach [77] and used the bias-corrected bootstrapping approach, including 95% bootstrap confidence intervals using 5,000 bootstrap samples in Mplus version 7.4 software [78]. Unstandardized coefficient estimates for the model were presented in Table 2. After controlling gender, age, education, and tenure, inclusive leadership was positively related to employee innovative behavior (β = 0.339**, SE = 0.081, p <0.01, 95%CI = [0.183, 0.503]). Because the CI did not contain zero. Thus, Hypothesis 1 was supported, indicating that when employees perceived more inclusive leadership, they demonstrated more innovative behavior. Inclusive leadership was positively related to POS (β = 0.843**, SE = 0.068, p <0.01, 95%CI = [0.710, 0.977]), supporting Hypothesis 2. This result indicated that when employees perceived more inclusive leadership, they experienced more POS. POS was positively related to employee innovative behavior (β = 0.244**, SE = 0.068, p <0.01, 95%CI = [0.112, 0.381]), supporting Hypothesis 3. This result demonstrated that when employees perceived more organizational support, they demonstrated more innovative behavior. Inclusive leadership was positively related to employee innovative behavior through POS (β = 0.206**, SE = 0.059, p <0.01, 95%CI = [0.092, 0.327]). The result indicated that POS mediated the relationship between inclusive leadership and employee innovative behavior.

**Table 2:** Unstandardized coefficients of the model.

Effects	Hypothesis	Estimate	SE	95% confidence intervals
**Direct effect**	Inclusive leadership → Employee innovative behavior	0.339**	0.081	[0.183, 0.503]
	Inclusive leadership → POS	0.843**	0.068	[0.710, 0.977]
	POS → Employee innovative behavior	0.244**	0.069	[0.112, 0.381]
**Indirect effect**	Inclusive leadership → POS → Employee innovative behavior	0.206**	0.059	[0.092, 0.327]

Note: N = 226,

** p <0.01,

* p <0.05

## Conclusion and discussion

In this study, we aimed to investigate how and why inclusive leadership influenced employee innovative behavior based on organizational support theory and social exchange theory. Consistent with organizational support theory, we found that inclusive leadership was positively related to employee innovative behavior and POS mediated the relationship between inclusive leadership and employee innovative behavior. That is, when employees perceived that leaders showed more inclusiveness to their new ideas, technologies, and processes, they perceived being more valued and cared about by the organization and thus, increased their innovative behavior.

### Theoretical implications

The current study made several important theoretical contributions. First, the findings suggested that inclusive leadership had an important effect on employee innovative behavior, which was consistent with research demonstrating the importance of supervisory support in innovative behavior [5,51,75]. This empirical work addressed important gaps in the innovation literature with respect to supportive determinants of employee’ innovative behavior [72]. Inclusive leadership was likely to act as an innovation–facilitating force. Inclusive leadership promoted employee innovative behavior by increasing POS and encouraging employees intellectually to bring forth alternative ways to solve existing problems or improve existing procedures.

This research also advanced the inclusive leadership literature by complementing the classic social exchange theory with fresh insights from the organizational support theory framework [50], thereby broadening its theoretical scope to account for support-based outcomes that hinge on employees’ POS. Although the concept of inclusive leadership has received increasing attention in recent years, inclusive leadership remained a new concept without consensus on the nature of the construct or its theoretical underpinnings. This lack of theoretical and practical consensus hampered the utility of inclusive leadership [79]. Our study was one of few studies to investigate the influence of inclusive leadership on POS and employee innovative behavior, which responded to the view that “the inclusion construct and its underlying theoretical basis need greater development” [79] and “much work remains to be done to advance theory related to our understanding of inclusive leadership” [18].

Second, our results highlighted the role of POS as a mediator for the relationship between inclusive leadership and employee innovative behavior. This finding contributed to the literature in that it showed POS as a mediator through which inclusive leadership influenced employee innovative behavior. In the past research, studies had not yet explored the mechanism underlying the relationship between inclusive leadership and employee innovative behavior from the perspective of organizational support. The current study complemented previous research by revealing how inclusive leadership increased employee innovative behavior through POS. Our findings were consistent with a view from Eisenberger, Fasolo, & Davislamastro’s saying that “perceived support might be associated with constructive innovation” [55].

Third, support for the positive effect of inclusive leadership on employee innovative behavior proposed an important role in addressing determinants of innovation. Advancing employee innovative behavior were critical to organization’s competitiveness and long-term success. Although an organization’s employees had idiosyncratic attributions and perceptions of leadership styles, our findings illustrated how an organization and its leaders can advance employee innovative behavior through inclusive leadership.

### Practical implications

In a dynamic environment, as organizational competiveness relied on employee innovative behavior, it was vital to identify how leaders can stimulate their follower innovation [51]. The theoretical model proposed in this paper could inform managers of how to improve employee innovative behavior. As mentioned, creativity/innovation was risky, requiring employees to change and act differently and leaders to tolerate and accept deviation from conventional practices. Since inclusive leadership was demonstrated to promote employee innovative behavior in this way, managers should develop skills of inclusive leadership in order to encourage employees engage in innovative behavior more. We suggested that leadership training programs could help leaders realize the importance of inclusiveness, openness and quip them with necessary skills to provide support for employees.

Moreover, the finding that POS mediated the relationship between inclusive leadership and employee innovative behavior suggested the need of paying attention to the underlying mechanism through which inclusive leadership stimulated followers’ innovative behavior. Managers should consider ways such as showing openness and inclusiveness to employees’ new ideas, technologies, and products, and valuing their efforts to increase employees’ POS. Furthermore, managers may offer other kinds of support, such opportunities, resources, and autonomy to employees to stimulate more innovative behavior.

Finally, given that people naturally tended to maintain their status quo, it was of critical importance to identify factors that could help employees to overcome this tendency and engage in more innovative behavior. Our findings suggested that inclusive leadership was a driver of employee innovative behavior.

### Limitations and future research

Several limitations of this study should be mentioned. While mentioning the study’s limitations, we simultaneously suggested directions for future research. The first limitation was that the sample size from one organization is small, which may reduce generalization, thus limiting the use of results pertaining to significant relationships. The current findings may provide conservative estimates of hypotheses testing, suggesting potentially stronger effects in industries where innovation are much higher (e.g., technology). Future research could advance our knowledge by replicating this study’s results across diverse industries using a larger sample size.

Second, the current study explored only one mechanism between inclusive leadership and employee innovative behavior, although other mechanisms may exist (e.g., psychological empowerment). Furthermore, it could be helpful investigate boundary conditions that might influence the relationship between inclusive leadership and innovation. Future research could examine other types of employee performance such as task performance.

## Supporting information

S1 Questionnaire Questionnaire I-III. (DOCX)

S1 Dataset (SAV)
