# Supplementary material for: Impact of inclusive leadership on employee innovative behavior: Perceived organizational support as a mediator
Source: PLoS One. 2019 Feb 28;14(2):e0212091. doi: 10.1371/journal.pone.0212091 (PMC6395022; doi:10.1371/journal.pone.0212091)
Supplement: S1 Questionnaire — (DOCX) [file pone.0212091.s001.docx]

**QUESTIONNAIRE I (Employee T1)**

This questionnaire is designed to understand the leadership and the support that you perceived of your organization. Please do not skip any of the questions as some of them appearing to the redundant with a purpose. All your responses will be held **strictly confidential**. Under no circumstance will your answers be made available to anyone except the research. And, your answers will only be used in an **aggregated and anonymous** fashion in all future reports.

**SECTION I:** The following items describe the leadership of your supervisor. Please indicate to what extent you agree with the following statement by circling the appropriate number on the rating scale provided.

| **Please indicate to what extent you agree with the following statements** | **Totally disagree** | **Disagree** | **Neutral** | **Agree** | **Totally agree** |
| --- | --- | --- | --- | --- | --- |
| 1. My supervisor is open to hearing new ideas | 1 | 2 | 3 | 4 | 5 |
| 2. My supervisor is attentive to new opportunities to improve work processes | 1 | 2 | 3 | 4 | 5 |
| 3. My supervisor is open to discuss the desired goals and new ways to achieve them | 1 | 2 | 3 | 4 | 5 |
| 4. My supervisor is available for consultation on problems | 1 | 2 | 3 | 4 | 5 |
| 5. My supervisor is an ongoing ‘presence’ in this team - someone who is readily available | 1 | 2 | 3 | 4 | 5 |
| 6. My supervisor is available for professional questions I would like to consult with him/her | 1 | 2 | 3 | 4 | 5 |
| 7. My supervisor is ready to listen to my requests | 1 | 2 | 3 | 4 | 5 |
| 8. My supervisor encourages me to access him=her on emerging issues | 1 | 2 | 3 | 4 | 5 |
| 9. My supervisor is accessible for discussing emerging problems | 1 | 2 | 3 | 4 | 5 |

**SECTION II: RESPONDENT’S PERSONAL BACKGROUND INFORMATION**

- Age □below 25 □26-35 □36-45 □46-55 □55 or above
- Gender □male=0 □female=1
- Education □high school □college degree □bachelor degree □graduate degree
- Work experience How long have you worked?

□less than 1 year □1-3 years □4-6 years □7-10 years □more than 11 years

**QUESTIONNAIRE II (Employee T2)**

This questionnaire is designed to understand the leadership and the support that you perceived of your organization. Please do not skip any of the questions as some of them appearing to the redundant with a purpose. All your responses will be held **strictly confidential**. Under no circumstance will your answers be made available to anyone except the research. And, your answers will only be used in an **aggregated and anonymous** fashion in all future reports.

The following items describe your perceived organizational support. Please indicate to what extent you agree with the following statement by circling the appropriate number on the rating scale provided.

| **Please indicate to what extent you agree with the following statements** | **Totally disagree** | **Disagree** | **Neutral** | **Agree** | **Totally agree** |
| --- | --- | --- | --- | --- | --- |
| 1. My organization cares about my opinions. | 1 | 2 | 3 | 4 | 5 |
| 2. My organization cares about my well-being. | 1 | 2 | 3 | 4 | 5 |
| 3. My organization appreciates any extra effort from me. | 1 | 2 | 3 | 4 | 5 |
| 4. My organization would ignore any complaint from me. | 1 | 2 | 3 | 4 | 5 |
| 5. Even if I did the best job possible, my organization would fail to notice. | 1 | 2 | 3 | 4 | 5 |
| 6. My organization cares about my general satisfaction at work. | 1 | 2 | 3 | 4 | 5 |
| 7. My organization shows very little concern for me. | 1 | 2 | 3 | 4 | 5 |
| 8. My organization takes pride in my accomplishments at work. | 1 | 2 | 3 | 4 | 5 |

**QUESTIONNAIRE III (Supervisor T2)**

This questionnaire is designed to test your subordinates’ innovative behavior in your team. Please do not skip any of the questions as some of them appearing to the redundant with a purpose. All your responses will be held **strictly confidential**. Under no circumstance will your answers be made available to anyone except the research. And, your answers will only be used in an **aggregated and anonymous** fashion in all future reports.

The following items describe your subordinates’ innovative behavior. Please indicate to what extent you agree with the following statement by writing the appropriate number for the right employee ***(1 = Totally disagree, 2 = Disagree, 3 = Neutral, 4 = Agree, 5 = Totally agree)***.

| **Please indicate to what extent you agree with the following statements** | **Employee A** | **Employee B** | **Employee C** | **Employee D** | **Employee E** |
| --- | --- | --- | --- | --- | --- |
| 1. He/she always creating new ideas for difficult issues |  |  |  |  |  |
| 2. He/she always searching out new working methods, techniques, or instruments |  |  |  |  |  |
| 3. He/she always generating original solutions for problems |  |  |  |  |  |
| 4. He/she always mobilizing support for innovative ideas |  |  |  |  |  |
| 5. He/she always acquiring approval for innovative ideas |  |  |  |  |  |
| 6. He/she always making important organizational members enthusiastic for innovative ideas |  |  |  |  |  |
| 7. He/she always transforming innovative ideas into useful applications |  |  |  |  |  |
| 8. He/she always introducing innovative ideas into the work environment in a systematic way |  |  |  |  |  |
| 9. He/she always evaluating the utility of innovative ideas |  |  |  |  |  |
